# Fermented Rice Bran Mitigated the Syndromes of Type 2 Diabetes in KK-*A^y^* Mice Model

**DOI:** 10.3390/metabo14110614

**Published:** 2024-11-11

**Authors:** Afifah Zahra Agista, Ami Kato, Tomoko Goto, Takuya Koseki, Akira Oikawa, Yusuke Ohsaki, Michiko Yamaki, Chiu-Li Yeh, Suh-Ching Yang, Ardiansyah, Slamet Budijanto, Michio Komai, Hitoshi Shirakawa

**Affiliations:** 1 Laboratory of Nutrition, Graduate School of Agricultural Science, Tohoku University, Sendai 980-8572, Japan; agista@g-mail.tohoku-university.jp (A.Z.A.); am.kato@g-mail.tohoku-university.jp (A.K.); tgoto@mgu.ac.jp (T.G.); yusuke.ohsaki.a4@tohoku.ac.jp (Y.O.); michio.komai.c2@tohoku.ac.jp (M.K.); 2 Faculty of Agriculture, Yamagata University, Tsuruoka 997-8555, Japan; tkoseki@tds1.tr.yamagata-u.ac.jp; 3 Graduate School of Agriculture, Kyoto University, Kyoto 606-8224, Japan; oikawa.akira.7j@kyoto-u.ac.jp; 4 RIKEN Center for Sustainable Resource Science, Yokohama 230-0045, Japan; 5 International Education and Research Center for Food Agricultural Immunology, Graduate School of Agricultural Science, Tohoku University, Sendai 980-8572, Japan; 6 Department of Home Economics, Division of Health and Nutrition, Tohoku Seikatsu Bunka University, Sendai 981-8585, Japan; m-yamaki@g-mail.tohoku-university.jp; 7 School of Nutrition and Health Sciences, Taipei Medical University, Taipei 11031, Taiwan; clyeh@tmu.edu.tw (C.-L.Y.); sokei@tmu.edu.tw (S.-C.Y.); 8 Department of Food Technology, Universitas Bakrie, Jakarta 12920, Indonesia; ardiansyah.michwan@bakrie.ac.id; 9 Faculty of Agricultural Engineering and Technology, IPB University, Bogor 16680, Indonesia; slametbu@apps.ipb.ac.id

**Keywords:** *Aspergillus kawachii*, diabetes, fermented rice bran, lactic acid bacteria

## Abstract

**Background:** Diabetes is a devastating disease that causes millions of deaths. Fermented rice bran (FRB), made by fermenting rice bran with *Aspergillus kawachii* and a mixture of lactic acid bacteria, was hypothesized to b able to improve diabetes-related symptoms. This study aimed to investigate the effects of FRB supplementation in mitigating type 2 diabetes symptoms and identifying FRB bioactive compounds. **Methods**: In this study, KK-*A^y^* mice (4 w.o. male) were used as a model for type 2 diabetes. Mice were divided into three different groups. The first group received a control diet, the second received a 12.5% non-fermented rice bran (RB) supplemented diet, and the last group was fed a 12.5% FRB-supplemented diet. Supplementation was done for 4 weeks. **Results**: FRB supplementation lowered the blood glucose level, OGTT, HOMA-IR, total cholesterol, liver RAGE protein, and *glucokinase* in KK-*A^y^* mice. Metabolome analysis of RB and FRB showed that fermentation increased bioactive compounds in rice bran, such as GABA, L-theanine, and carnitine. It also increased the levels of various free amino acids while converting some amino acids such as arginine, tyrosine, and tryptophan into other metabolites. **Conclusions**: This research showed the potency of FRB supplementation as a preventive agent against type 2 diabetes.

## 1. Introduction

Diabetes is one of the leading causes of death and disability, and its prevalence has continued to increase all over the world. Around 529 million people in the world were diagnosed with diabetes in 2021. This number mainly consists of the number of people living with type 2 diabetes, which contributes to 96% of the total number [1]. The onset of diabetes has also been associated with several comorbidities, such as chronic kidney diseases, cardiovascular diseases, retinopathy, sarcopenia, and others [2,3,4]. Patients who have been diagnosed with type 2 diabetes were found to have shorter life expectancy, depending on the age of their diagnosis [5,6].

Treatment for type 2 diabetes commonly involves a combination of lifestyle management and pharmaceutical treatment. Pharmaceutical treatments that are used to treat type 2 diabetes include insulin sensitizers, insulin-promoting agents, α-glucosidase inhibitors, incretin-based therapies, and sodium-glucose co-transporter 2 inhibitors. However, pharmaceutical treatment can often bring about some adverse side effects, depending on the treatment which is used. Some known side effects are weight gain, hypoglycemia, porphyria, diarrhea, urinary tract infections, risk of bone fractures, and risk of renal impairment [7,8,9,10,11]. Therefore, preventing the onset of type 2 diabetes and regulating its symptoms are much more desirable. Some bioactive compounds, such as various polyphenols and flavonoids, have been acclaimed to prevent the onset of type 2 diabetes [7,12,13]. Other bioactive compounds and natural products that exert similar anti-diabetic action are also highly sought. Fermented foods have been hailed as functional food products with various health benefits. One of the health benefits of fermented food is its anti-diabetic activity. A lactic-acid-bacteria-fermented food product was reported to maintain blood glucose levels and improve glucose tolerance in rats fed a hypercaloric diet [14]. Food products fermented by fungi such as *Aspergillus oryzae*, *Aspergillus luchuensis*, *Rhizopus oligosporus*, and *Rhizopus oryzae* were suggested to be able to alleviate dyslipidemia and hyperglycemia [15].

Rice is a popular staple food choice for many people in the world. Rice bran (RB) is a by-product of the rice milling industry; it is usually used as animal feed and therefore has low value. However, RB is known to contain various bioactive compounds such as γ-oryzanol, ferulic acid, phytosterols, triterpene alcohols, and tocotrienols [16]. RB was proven to have plasma (or serum) lipid-lowering, antioxidant, anti-inflammatory, and anti-diabetic activities [17]. Fermentation of RB with *Aspergillus kawachii* (*Aspergillus luchuensis* mut. *kawachii*) followed by a mixture of *Levilactobacillus brevis* (formerly *Lactobacillus brevis*), *Lacticaseibacillus rhamnosus* (formerly *Lactobacillus rhamnosus*), and *Enterococcus faecium* was suggested to enhance its nutritional value. *Aspergillus kawachii* is a fungus generally recognized as safe and often used in the shochu brewing industry in the saccharification process of starches such as barley, rice, and sweet potatoes [18]. *A. kawachii* fermentation also tends to produce citric acid, preventing undesired microorganisms’ growth. Lactic acid bacteria, however, are tolerant of low pH, and the combination of lactic acid bacteria and citric acid was reported to prevent the growth of *Salmonella* Typhimurium and *Escherichia coli* O157:H7 [19]. Alauddin et al. [20] found that this fermented rice bran (FRB) was able to reduce blood pressure, improve glucose tolerance and insulin sensitivity, and increase serum insulin levels in stroke-prone spontaneously hypertensive rats (SHRSPs). FRB was also reported to be able to ameliorate streptozotocin-induced sarcopenia in Sprague Dawley rats [21]. Consequently, FRB will likely be able to lessen the symptoms of type 2 diabetes.

This study investigates the effect of FRB supplementation in KK-*A^y^* mice as an animal model of type 2 diabetes. It also observes FRB supplementation’s effect on maintaining lipid profiles in diabetic conditions. Furthermore, this study also explored the metabolites found in FRB that may play vital roles in its health-enhancing capacity.

## 2. Materials and Methods

### 2.1. Materials and Reagents

This study used chemical reagents such as methanol, ethanol, chloroform, and H_2_SO_4_ from Wako Pure Chemical Industries, Ltd. (Osaka, Japan). The grade of the reagents was adjusted to their usage. Mass spectrophotometry and HPLC-grade reagents were used in metabolome analysis, while guaranteed reagent-grade reagents were used in other analyses. Fermentation starters for *A. kawachii* and lactic acid bacteria were obtained from Akita Konno Co. Ltd. (Akita, Japan) and Sunstar Inc. (Osaka, Japan), respectively.

### 2.2. Fermented Rice Bran Preparation

The FRB preparation process was described in a previous study [20]. Firstly, partially defatted rice bran was mixed with water (1:1) and steamed at 121 °C for 20 min. After the steamed mixture cooled down to 30 °C, *A. kawachii* starter (10^6^ spores/g of RB) was inoculated. The inoculated mixture was then incubated in a shallow, rectangular vat vessel (solid-state fermentation) in an incubator at 30 °C for 44 h. The solid-state culture obtained was designated as rice bran koji [22]. The moisture contents of RB and rice bran koji were analyzed using the dry heat method at 105 °C for 5 h. The produced rice bran koji was then mixed with rice powder (dry weight 2:1) and water (4-fold the total amount of the total mixture), saccharified at 56 °C for 12 h, and cooled before the lactic acid bacteria culture (consisting of a mix of *L. brevis*, *L. rhamnosus*, and *E. faecium* in the concentration of 0.01% *w*/*w*) was added. This mixture was then incubated at 37 °C overnight. The fermented mixture was then heated at 85 °C for 15 min. Non-fermented rice bran was prepared similarly without the addition of microorganisms. The product was then filtered, and a portion of the filtrate was used for the metabolomic analysis. The remaining filtrate was further lyophilized and stored at −30 °C. These lyophilized products were used in diet supplementation (Table 1).

### 2.3. Animal Experiment

Male KK-*A^y^*/TaJcl mice (CLEA Japan, Inc., Tokyo, Japan) aged 3 weeks were purchased and used in these experiments. Twenty-four mice were used (8 mice in the control group, 7 in the RB groups, and 9 in the FRB groups). The KK-*A^y^* mouse is a hybrid between the KK and *A^y^* mice. The KK mouse was initially established as a diabetic model, and the *A^y^* gene was then introduced as this gene was hypothesized to be responsible for the hyperglycemic obese syndrome that the yellow-coated *A^y^* mouse showed. The hybrid between these two mice shows severe diabetes mellitus syndrome, which includes glycosuria, hyperglycemia, and decreased ability to assimilate glucose. The fed glucose level of this mouse strain ranged from 100 to 600 mg/dL, and its fasting blood glucose level was reported to reach more than 100 mg/dL. Thirty minutes after glucose administration, its blood glucose level reached more than 500 mg/dL [23].

Mice were housed in a pathogen-free environment at approximately 23 ± 3 °C, relative humidity 55 ± 10%, and lighting cycle 12 h/d. Mice had free access to diet and drinking water. After one week of habituation, mice were divided into the control, RB, and FRB groups. Control groups were given a diet based on the AIN-93G composition, RB groups were given the same diet supplemented with rice bran (12.5%), and FRB groups were given a diet supplemented with fermented rice bran (12.5%). The composition of the diets is displayed in Table 1. Mice were maintained on the same diet for 4 weeks, and afterward, they were euthanized. This experiment was approved by the Animal Research-Animal Care Committee of Tohoku University, and the corresponding ethical approval code is 2017Ag-021.

### 2.4. Serum and Liver Biochemical Analysis

Serum glucose levels were measured weekly using a glucose analyzer StatStrip Express (Nova Biomedical Co., Waltham, MA, USA). Briefly, the mouse tail was wounded with a scalpel, and the tip of the StatStrip was used to pick up the blood obtained from the wound. The strip was then attached to a machine, which then measured the glucose level. Blood insulin levels were measured using a mouse insulin assay kit (Morinaga BioScience, Inc., Yokohama, Japan). After euthanizing, mouse serum was mixed with guinea pig anti-insulin serum and incubated overnight at 4 °C. Enzyme-labeled anti-guinea pig IgG antibody solution was added. The mixture was again incubated for an hour at room temperature. Enzyme substrate was added following incubation, and after 30 min, a stop solution was mixed, and the absorbance of each sample was measured at 450 nm with 630 nm reference wavelength.

Total cholesterol (TC) and triglycerides (TGs) were measured by utilizing enzymatic colorimetric methods (Wako Pure Chemical Industries, Ltd., Osaka, Japan). These assays were performed according to the manufacturer’s instructions. A serum sample was mixed with the chromogen reagent as it was. This mixture was then incubated for 5 min at 37 °C, and the absorbance of each sample was then taken at 600 nm. A frozen liver sample was homogenized with 1 mL methanol. Two mL of chloroform was added, and the sample was further homogenized, followed by another 2 mL of methanol/chloroform (1:2). The homogenate was filtered, and 1 mL of 0.05% H_2_SO_4_ was added to the filtrate. After centrifugation (3000 rpm, 4 °C, 10 min), the organic layer was taken, and 1 mL of chloroform containing 1% Triton x-100 was added. The mixture was then evaporated with a rotary evaporator at 1800 rpm, 40 °C, until the solvent had completely disappeared. Afterward, 500 µL of MilliQ water was added, and this sample was used similarly to serum samples.

### 2.5. Enzyme-Linked Immunosorbent Assay (ELISA)

Liver tissues were digested, dissolved in lysis buffer, and diluted appropriately according to different analyses. Enzyme-linked immunosorbent assays were then conducted by using ELISA kits to measure the liver’s receptor for advanced glycation end products, also known as RAGE or AGER (R&D Systems, Minneapolis, MN, USA), CML (Cell Biolabc Inc., San Diego, CA, USA), and TNFα (Diaclone, Besançon, France). The insulin level in blood was also analyzed using an ELISA kit per the manufacturer’s instruction (Morinaga Institute of Biological Science, Inc., Kanagawa, Japan).

### 2.6. Oral Glucose Tolerance Test (OGTT)

An OGTT was conducted after mice were kept in fasting condition for 16 h. Glucose (1.8 g/kg body weight) was administered via oral gavage, and blood samples were collected from the tail vein at 0, 30, 60, and 120 min after glucose was given. The glucose and insulin levels in plasma were then analyzed from the collected blood samples. The area under the curve (AUC) was calculated using incremental blood glucose response areas and ignoring the area below the baseline. The homeostasis model assessment of insulin resistance index (HOMA-IR) was calculated based on the concentration of glucose and insulin in mice serum, obtained after mice were euthanized. HOMA-IR was calculated using the following formula: HOMA-IR = fasting insulin concentration (µL/mL) × fasting glucose concentration (mg/dL)× 26/405.

### 2.7. Quantitative Reverse Transcriptase-Mediated Polymerase Chain Reaction (qRT-PCR)

Isogen, a guanidine isothiocyanate-based reagent, was used to isolate RNA from mouse tissues. After purification, the obtained RNA was used as a template to synthesize cDNA. The produced cDNA was then subjected to qRT-PCR. Target cDNAs were amplified with SYBR Premix Ex Taq solution (Takara Bio, Otsu, Japan) and gene-specific primers. The list of primers is displayed in Supplementary Table S1. The Applied Biosystems 7300 Real Time PCR System (Foster City, CA, USA) was used in this process. The amounts of mRNA expression were then normalized in reference to the obtained amount of Eukaryotic translation elongation factor 1α1 (Eef1α1) mRNA.

### 2.8. Capillary Electrophoresis Mass Spectrometry (CE-MS)

CE-MS analysis was performed according to a method previously described by Oikawa et al. [24]. Before the metabolomic analyses, hydrophobic and high-molecular-weight compounds were removed by the preparative processes of liquid–liquid separation using chloroform and water and ultrafiltration using a 5 kDa cutoff filter, respectively, for the analysis of ionic metabolites. As previously reported, a comprehensive analysis of ionic metabolites was performed using CE-TOF MS [24]. Data describing the quantified or relative amounts of metabolites were integrated into one sheet. These data were standardized by subtracting the averages from each amount, dividing the averages from each amount, and dividing the resulting values by the standard deviations. The standardized data were using DrDMass (http://kanaya.naist.jp/DrDMASS/). This software was accessed online on 1 December 2017.

### 2.9. Statistical Analysis

Data were presented as means ± SE. The analysis was conducted using the Statcel 3 program (OMS publishing, Saitama, Japan) and MetaboAnalyst 6.0 (https://www.metaboanalyst.ca, accessed online on 24 July 2024). Data were analyzed using the Student’s *t*-test, one-way ANOVA, or two-way repeated measurement ANOVA. Tukey–Kramer was applied as a post hoc test. Significant differences between groups are denoted in each figure.

## 3. Results

### 3.1. FRB Supplementation Alleviates Diabetic Parameters

**Figure 1:**
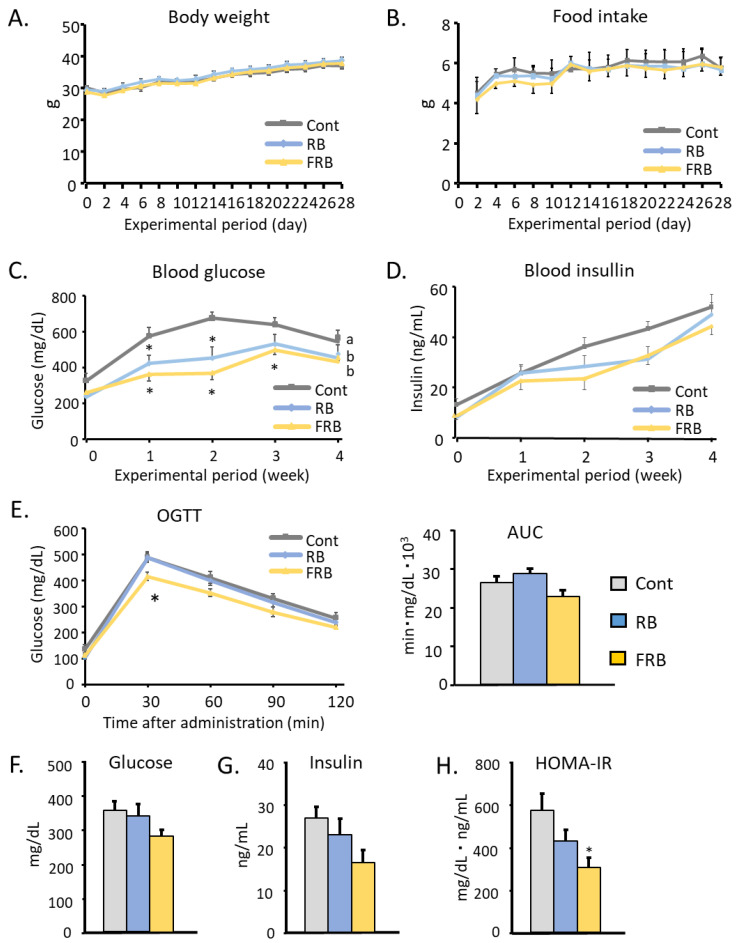
FRB supplementation alleviates diabetes symptoms. (**A**) Body weight (gram: g) and (**B**) food intake (g) from the control, RB, and FRB groups were observed for 28 days. (**C**) Blood glucose and (**D**) blood insulin were measured weekly for 4 weeks. (**E**) Oral glucose tolerance test and the calculated area under the curve. At the end of the experiment, the levels of (**F**) blood glucose, (**G**) insulin, and (**H**) homeostatic model assessment of insulin resistance were measured. Data are presented as means with error bars representing SE, n = 7–9, Dunnet, * *p* < 0.05, a, b, represent statistically different groups at the indicated *p*-value.

FRB supplementation did not affect the body weight or the food intake of KK-*A^y^* mice (Figure 1A,B). Nevertheless, Figure 1C demonstrates that mouse blood glucose levels significantly decreased throughout the experiment after receiving RB or FRB supplementation (Figure 1C). These changes persisted until the end of the experiment period. However, no difference was found in mouse plasma insulin levels (Figure 1D). The plasma glucose level of the FRB group after the oral glucose tolerance test (OGTT) was conducted was significantly reduced after 30 min since administration (Figure 1E). The FRB group was not shown to have statistical differences in its glucose and insulin levels; however, the level of HOMA-IR at the end of the experiment period was found to be significantly decreased compared to the other groups (Figure 1F–H).

**Figure 2:**
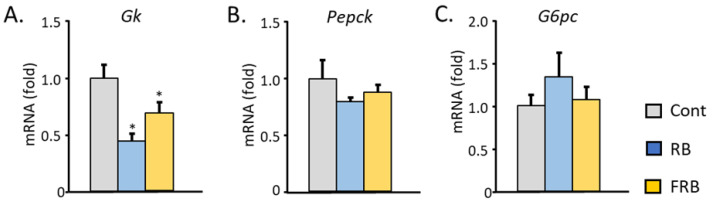
FRB and RB supplementation (12.5%) were able to lower hepatic glucokinase (*Gk*) mRNA. The mRNA levels of (**A**) *Gk*, (**B**) *Pepck*, and (**C**) *G6pc* are illustrated. Data are presented as means with error bars representing SE, n = 7–9, Dunnet, * *p* < 0.05.

Both RB and FRB groups have significantly lower glucokinase (Gk) mRNA expression (Gk, Figure 2A). Nevertheless, no difference was observed in the levels of Pepck (Phosphoenolpyruvate carboxykinase) and G6pc (Glucose-6-phosphatase) (Figure 2B,C).

### 3.2. Lipid Profile of KK-A^y^ Mice Was Improved Due to FRB Supplementation

**Figure 3:**
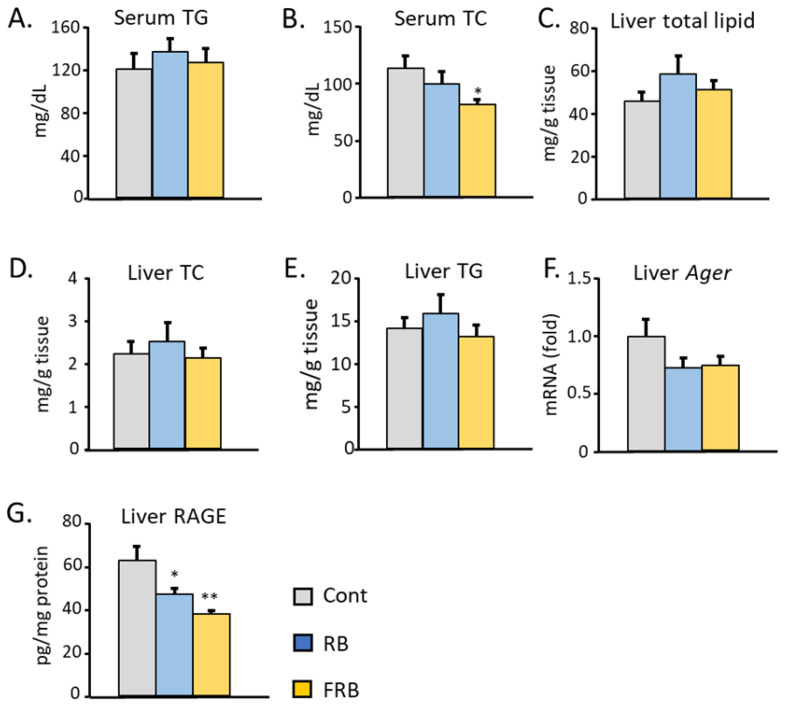
Supplementation of FRB affected lipid metabolism and glycation product receptor activation. (**A**) Triglycerides and (**B**) total cholesterol from blood were measured. (**C**) The levels of total lipids, (**D**) total cholesterol, and (**E**) triglycerides were measured. FRB supplementation lowers the expression of (**F**) *Ager* mRNA and (**G**) the level of RAGE in mouse livers. Data are presented as means with error bars representing SE, n = 7–9, Dunnet, * *p* < 0.05, ** *p* < 0.01.

Type 2 diabetes is often accompanied by abnormalities in lipid metabolism [25]. As shown in Figure 3A, we were not able to discern any differences in the serum level of total triglycerides due to the supplementation of FRB. On the other hand, FRB supplementation significantly reduced total cholesterol levels in serum (Figure 3B). While there was no difference in the levels of total lipids, triglycerides, and total cholesterol in the liver (Figure 3C–E), both RB and FRB treatments were found to slightly lower the mRNA expression of advanced glycation end products, also known as *Ager* (Figure 3F) and significantly decrease the level of RAGE protein in the liver (Figure 3G).

### 3.3. Metabolomics Analysis of RB and FRB

**Figure 4:**
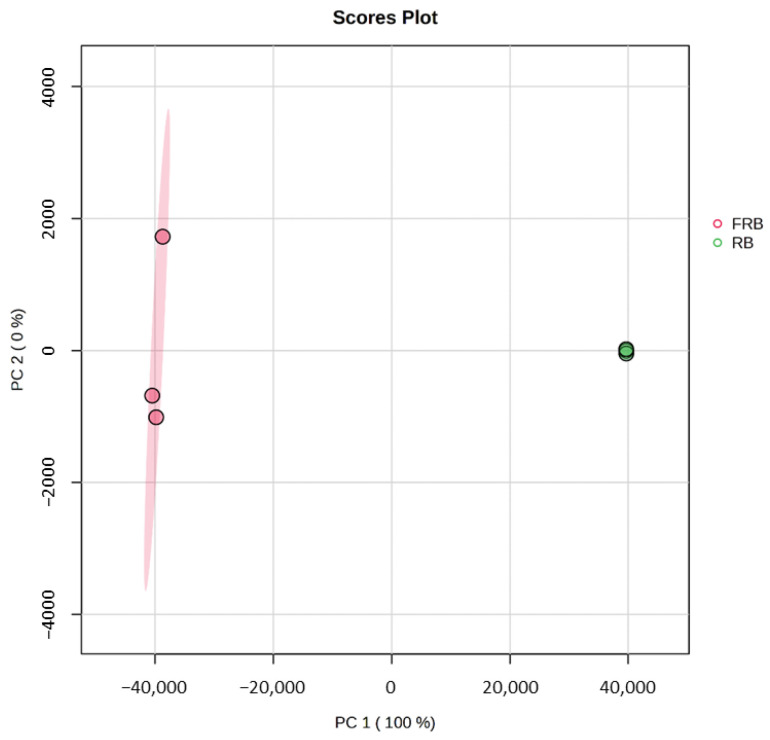
Principle component analysis on the effect of fermentation on rice bran.

**Figure 5:**
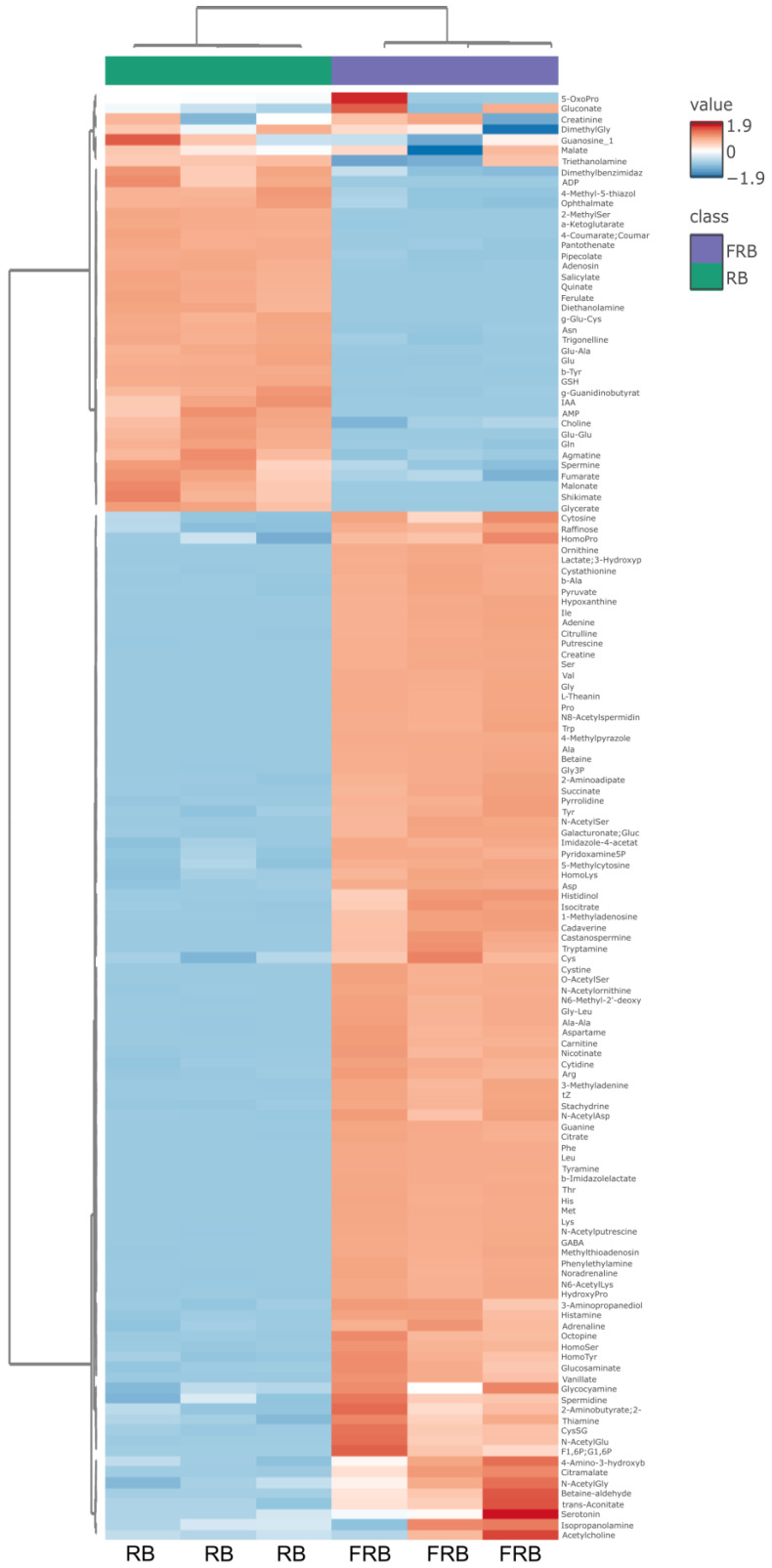
A comparison between metabolite contents in RB and FRB is displayed in a heat map.

Metabolomic analysis of RB and FRB was conducted to explore the differences between the bioactive compounds that arise in FRB due to fermentation. Figure 4 shows the principal component analysis (PCA) of metabolites from RB and FRB. The score plot from this analysis showed that FRB differs from RB, especially on the PC1 (100%). A heat map of 135 metabolites identified in either RB or FRB was generated and is displayed in Figure 5.

### 3.4. Bioactive Compounds in FRB

**Figure 6:**
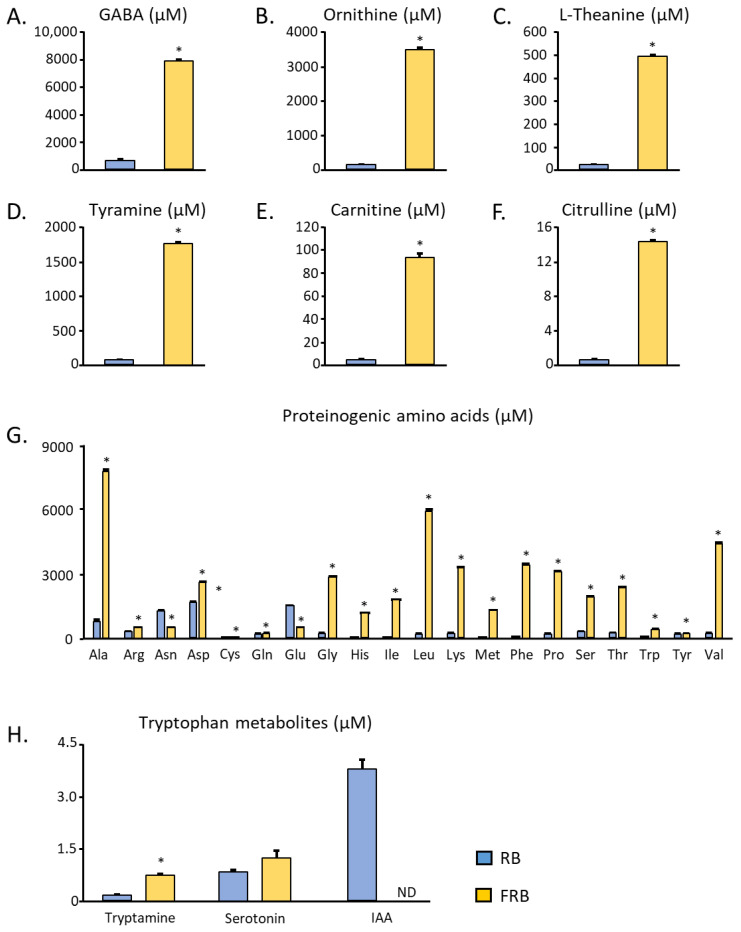
Bioactive compound comparison in RB and FRB. (**A**) GABA, (**B**) ornithine, (**C**) L-theanine, (**D**) tyramine, (**E**) carnitine, and (**F**) citrulline were found to be increased due to fermentation. (**G**) The levels of proteinogenic amino acids in RB are mostly augmented. (**H**) Tryptophan metabolite alteration due to fermentation. ND: the substance was not detected. Data are presented as means with error bars representing SE, n = 3, *t*-test, * *p* < 0.05.

Among the 135 metabolites identified, we have isolated the data for several compounds known to have some functional properties. This list of isolated bioactive compounds includes GABA, tyramine, ornithine, L-theanine, carnitine, and citrulline (Figure 6A–F). Fermentation was found to increase the amount of these bioactive compounds in RB. Furthermore, to evaluate whether the fermentation process affects the bioavailability of the nutrients in RB, we have isolated twenty proteinogenic amino acids. Figure 6G shows that most proteinogenic amino acids increase after fermentation, except asparagine, glutamine, and glutamic acid. Alanine, followed by leucine and valine, are the amino acids found in the highest amounts in RB.

Previous research has suggested that FRB’s tryptophan content and its various metabolites may contribute to FRB’s anti-inflammatory effect. Therefore, we also investigated the amount of tryptophan metabolites in this study. Fermentation was found to increase the amount of tryptamine, maintain the level of serotonin, and decrease the amount of indole-3-acetic acid in RB (Figure 6H).

## 4. Discussion

Fermentation of rice bran with *Aspergillus kawachii* followed by a mixture of *Lactobacillus brevis*, *Lactobacillus rhamnosus*, and *Enterococcus faecium* was previously shown to increase the phenolic compound and dietary fiber contents of RB. Alauddin et al. [20] insinuated that these compounds played a key role in FRB’s capacity to improve glucose tolerance, insulin sensitivity, and decreased serum insulin levels in SHRSPs. This notion was parallel with our results, which further proved that FRB was also able to lower the level of blood glucose and OGTT in KK-*A^y^* mice as a model for type 2 diabetes.

A high blood glucose level often leads to the formation of advanced glycation end products (AGEs). AGEs play a significant role in diabetes development and its complications. This role is mainly exerted through the receptors of advanced glycation end products (RAGEs) [26]. FRB supplementation was found to decrease the level of RAGEs in mice’s livers. The accumulation of AGE species can also lead to dyslipidemia in diabetic rats. Yuan et al. [27] mentioned that CML, one of the AGEs, disturbs the cholesterol feedback mechanism in the kidney of diabetic rats by increasing HMG-CoA reductase-mediated cholesterol synthesis and LDL receptor-mediated cholesterol uptake. Since nearly 65% of diabetes-related mortality is caused by heart disease and stroke, regulating the level of cholesterol in patients with diabetes is of utmost importance [28]. FRB supplementation lowered mice’s total cholesterol levels compared to the other groups. Furthermore, dyslipidemia and hyperglycemia can also lead to oxidative stress that will decrease insulin resistance [29]. The FRB group was found to have a significantly lower level of HOMA-IR, suggesting that FRB was able to improve the insulin resistance of KK-*A^y^* mice. In this manuscript, we have also observed that FRB supplementation can decrease the mRNA expression of *Glucokinase*. Glucokinase, G6PC, and PEPCK are critical to maintaining blood glucose levels. The activity of glucokinase is known to be dependent on the blood glucose level [30]. In this study, the decline in glucokinase expression in the FRB group was suggested to be related to its lower blood glucose level. However, since reductions in the levels of both *G6pc* and *Pepck* were not observed, FRB supplementation might not directly affect the gluconeogenesis system.

Fermentation was shown to increase the levels of GABA, L-theanine, and carnitine. These compounds are known to have some favorable effects in improving diabetes-related conditions. GABA is a well-known neurotransmitter that is naturally produced by pancreatic β-cells. GABA enhanced β-cell proliferation, reduced cell death, and increased insulin production while decreasing glucagon levels [31]. GABA intervention in rats receiving streptozotocin treatment and a high-fat diet decreased their blood glucose level and increased their plasma insulin level [32]. L-theanine is a compound commonly found in tea. The consumption of L-theanine has been reported to correlate with a lower risk of type 2 diabetes in the general Japanese population [33]. In healthy SD rats, L-theanine regulated glucose through insulin and the AMPK pathway. L-theanine also promotes fatty acid oxidation, suppressing lipid and cholesterol synthesis via the LKB1-related pathway [34,35]. Carnitine supplementation has been reported to improve insulin resistance [36] and improve various indicators in patients with impaired glucose tolerance, for example, reducing TGs, TC, LDL-C, and HbA1c [37,38,39,40].

Arginine, ornithine, and citrulline are part of the arginase–urea pathway. Arginine catabolism into citrulline or ornithine was reported in several lactic acid bacteria fermentations [41,42,43,44]. This observation was in line with the results we obtained in this study. While there was a significant increase in the level of arginine in FRB, the difference was less drastic compared to some other amino acids. Instead, the arginine released by the fermentation process was possibly immediately converted into ornithine, and a small portion was metabolized into citrulline. It is also important to note that arginine, citrulline, and ornithine are also involved in nitric oxide (NO) synthesis. Arginine and citrulline can be used by NO synthase (NOS) in NO production. Meanwhile, arginase, which competes with NOS, will convert arginine into urea and ornithine. NO production is essential in regulating insulin secretion and blood glucose levels. It is also crucial in maintaining the vascular function of patients with diabetes [45,46]. Citrulline supplementation was shown to increase plasma NO levels and was indicated to improve insulin and blood glucose homeostasis [45,47,48]. The effect of ornithine on type 2 diabetes needs to be studied further.

Tyrosine is another amino acid in rice bran that may have been metabolized further by fermentation. Tyrosine is known to be metabolized by lactic acid bacteria to tyramine [49,50]. Our data showed that the amount of tyrosine barely increased, while the amount of tyramine greatly increased in FRB. This raises the possibility that the bulk of tyrosine in rice bran might have been transformed during the fermentation process into tyramine. It also needs to be mentioned that tyramine is a trace amine-associated receptor (TAAR) ligand. This receptor is known to regulate insulin secretion in pancreatic β-cells [51]. TAAR ligands other than tyramine might also contribute to the anti-diabetic properties of FRB. Tryptamine, for example, is also a ligand of TAAR.

We have previously identified tryptophan and its microbial metabolites, such as tryptamine, indole, and indole-3-acetic acid, as bioactive compounds that potentially play a crucial role in FRB’s anti-inflammatory capacity [52,53]. While the amounts of tryptophan and tryptamine were increased in this study, the combined compounds were nowhere close to some of the other amino acids. These differences might be explained by the differences in the lot number of rice bran material and the two stages of the fermentation process. There is also the possibility that fermentation converted tryptophan into other secondary metabolites not identified in the current method. Overall, the effect of each stage of rice bran fermentation on the bioavailability of protein, amino acids, and their metabolites needs to be further studied.

Our previous study also discovered that fermentation increased the level of total phenolic compounds from 6.5 g gallic acid equivalent per gram (g GAE/g) of rice bran to 8.6 g GAE/g fermented rice bran. While this manuscript did not extensively cover the effect of phenolic compounds and dietary fiber in ameliorating diabetic conditions, it is suggested that phenolic compounds and dietary fiber play a synergetic role as an adjunct to the other bioactive compounds in FRB. Due to the complex systems of bioactive compounds in FRB, it is difficult to identify the effect of each compound. We hypothesize that rice bran fermentation by *A. kawachii* and lactic acid bacteria may produce phenolic acids that may prevent oxidative stress and decrease RAGE activation. This process by itself will lead to improving mice’s glucose tolerance and insulin resistance. However, FRB is also shown to contain GABA, L-theanine, carnitine, ornithine, citrulline, and tyramine, which are known to increase insulin production and improve insulin sensitivity and glucose tolerance capacity. The effect of these bioactive compounds, in turn, will ameliorate the mice’s dyslipidemia and hyperglycemia symptoms. A study to identify the effects of each bioactive compound in FRB and its capacity to ameliorate the diabetic syndrome needs to be performed in the future. Moreover, a study on how fermented rice bran can be incorporated in food products is also necessary. Considering that efforts have been made to incorporate wheat bran into wheat products such as bread, pasta, noodles, and cookies [54], we hypothesized that incorporating FRB in similar products is also possible.

## 5. Conclusions

Four weeks of supplementations of rice bran fermented with *Aspergillus kawachii* and a mixture of lactic acid bacteria was able to lower the blood glucose level, OGTT, HOMA-IR, *Glucokinase*, serum total cholesterol, and liver RAGE in KK-A*^y^* mice. Fermentation increases GABA, L-theanine, and carnitine levels, which are known to have some favorable effects against diabetes. It also increased the bioavailability of various amino acids in rice bran. Fermentation further converted some amino acids such as arginine, tyrosine, and tryptophan into other metabolites such as ornithine, citrulline, tyramine, and tryptamine. This research showed that FRB supplementation may be used as a preventive treatment against type 2 diabetes.

## Figures and Tables

**Table 1:** Diet composition.

Ingredients	CON	RB	FRB
*tert*-Butylhydroquinone	0.0014	0.0014	0.0014
L-Cystine	0.26	0.26	0.26
Choline bitartrate	0.22	0.22	0.22
Vitamin mixture	0.88	0.88	0.88
Mineral mixture	4.31	3.06	3.06
Soybean oil	7.38	6.13	6.13
Cellulose	7.13	4.38	4.38
Sucrose	8.75	8.75	8.75
Casein	19.4	17.5	17.5
Corn starch	49.6	46.3	46.3
Distilled water	2.1	−	−
RB	−	12.5	−
FRB	−	−	12.5

## Data Availability

All data are provided within the manuscript.

## Supplementary Materials

The following supporting information can be downloaded at https://www.mdpi.com/article/10.3390/metabo14110614/s1, Table S1: List of primers.
