# Supplementary material for: Fermented Rice Bran Mitigated the Syndromes of Type 2 Diabetes in KK-Ay Mice Model
Source: Metabolites. 2024 Nov 11;14(11):614. doi: 10.3390/metabo14110614 (PMC11596254; doi:10.3390/metabo14110614)
Supplement: Supplementary file 1 [file metabolites-14-00614-s001.zip › metabolites-3203081-supplementary.pdf]

Table S1. Primer list

Gene Name	Forward/Reverse	Primer Sequence
Ager	Forward	TACCTTCTCCTGCAGTTTCAGC
	Reverse	CCCTGACTCGGAGTTGGATG
Gck	Forward	GTGGCAATGGTGAACGACAC
	Reverse	AATGTCGCAGTCGGTGACAG
G6pc	Forward	TTGTGCATTTGCTAGGAAGAGAAG
	Reverse	ATCTAAAGACCCAGGCATAACTGAAG
Pepck	Forward	GAGGACATTGCCTGGATGAAGTTT
	Reverse	TGGGTTGATGGCCCTTAAGT
